# Monoclonal Gammopathy of Clinical Significance (MGCS) and Related Disorders: A Review and the Role of Imaging

**DOI:** 10.3390/diagnostics14171907

**Published:** 2024-08-29

**Authors:** Ahmed O. El Sadaney, Anika Dutta, Joselle Cook, Francis I. Baffour

**Affiliations:** 1 Department of Radiology, Mayo Clinic, Rochester, MN 55905, USA; rabie.ahmed@mayo.edu (A.O.E.S.);; 2 Division of Hematology, Department of Internal Medicine, Mayo Clinic, Rochester, MN 55905, USA; cook.joselle@mayo.edu

**Keywords:** MGCS, MGUS, multiple myeloma, POEMS, amyloidosis

## Abstract

The term monoclonal gammopathy of clinical significance (MGCS) refers to a group of symptomatic monoclonal gammopathies that do not meet the diagnostic criteria for malignant plasma cell disorders, such as multiple myeloma or Waldenström macroglobulinemia. These symptoms are attributable to the paraneoplastic effects of monoclonal immunoglobulins that occur through diverse mechanisms. The presence of symptoms distinguishes MGCS from monoclonal gammopathy of undetermined significance, which lacks significant symptomatic presentation. The presentations of MGCS are manifold, adding to the diagnostic challenge. Clinical suspicion is key for accurate and timely diagnosis. Radiologic imaging can provide pivotal information to guide the diagnosis. In this review, we discuss MGCS from a radiology perspective and highlight pertinent imaging features associated with the disorders.

## 1. Introduction

The proliferation of monoclonal plasma cells leads to clinical conditions, ranging from malignancies, such as multiple myeloma (MM) and Waldenström macroglobulinemia, to nonmalignant entities like monoclonal gammopathy of undetermined significance (MGUS) [1]. When patients with MGUS exhibit unexplained symptoms, the condition is termed monoclonal gammopathy of clinical significance (MGCS). MGCS is characterized by a clonal proliferation of plasma cells, not meeting the threshold for MM (bone marrow < 10% plasmacytosis) and associated with clinically significant manifestations, commonly affecting the nervous, renal, and cutaneous systems [2,3,4]. Symptom constellations encompass neuropathies, nephropathies, dermatological disorders, endocrinopathies, and occasionally anasarca. The multitude and nonspecific nature of these symptoms pose diagnostic challenges, necessitating heightened clinical suspicion. This review will highlight those MGCS where imaging findings and radiologic assessment are critical to the diagnosis.

## 2. Pathophysiologic Classification Versus Organ Involvement Classification

The 5th edition of the WHO classification of hematolymphoid tumors focusing on lymphoid neoplasms proposed a lineage-based stratified classification that includes, for the first time, entities related to paraproteins that were previously not included in prior editions [5]. These include monoclonal gammopathy of renal significance (MGRS) and cold agglutinin disease [5]. The International Consensus Classification of small B-cell lymphoid neoplasms considers MGCS and MGRS as descriptive conditions under the larger MGUS umbrella [6]. Other approaches to classifying MGCS include pathophysiological classification based on the mechanisms through which immunoglobulins exert damage on organs [2]. In contrast, Dispenzieri proposed a clinically oriented classification based on organ involvement [2,7]. It is noteworthy that these disorders often involve multiple organs and may overlap or affect other systems.

## 3. MGCS with Neuropathic Involvement

Monoclonal protein disorders may result in various neurologic presentations, ranging from distal length-dependent sensory neuropathies to demyelinating disorders. The neuropathies may occur with multisystem involvement or may be the only predominant symptom. With the latter, the term monoclonal gammopathy of neurologic involvement may be used.

### 3.1. POEMS Syndrome

POEMS syndrome, named for its characteristic symptoms (polyradiculoneuropathy, organomegaly, endocrinopathy, monoclonal plasma cell disorder, and skin changes), is driven by cytokine effects, particularly vascular endothelial growth factor (VEGF).

The diagnosis of POEMS encompasses those described in the acronym as well as additional features such as PEST: papilledema, extravascular volume overload, sclerotic bone lesions, and thrombocytosis/erythrocytosis [8]. The diagnostic criteria involve a combination of mandatory major and minor criteria comprising clinical manifestations, laboratory, and imaging findings [9]. For a definitive diagnosis, two mandatory major criteria must be present: polyneuropathy, typically manifesting as a demyelinating sensorimotor polyneuropathy, and a monoclonal plasma cell proliferative disorder, often characterized by monoclonal gammopathy with lambda light chains [8]. Additionally, one other major criterion is required, which could be either histologically proven Castleman disease, sclerotic bone lesions identified on imaging studies, or elevated VEGF levels in the serum [8].

In addition to these major criteria, at least one minor criterion must be met. These minor criteria include organomegaly (such as hepatomegaly, splenomegaly, or lymphadenopathy), extravascular volume overload (evidenced by peripheral edema, pleural effusion, or ascites), endocrinopathy (with abnormalities in adrenal, thyroid, pituitary, gonadal, parathyroid, or pancreatic function), skin changes (such as hyperpigmentation, hypertrichosis, glomeruloid hemangiomas, extra-renal hemangiomas, white nails, or clubbing), papilledema (swelling of the optic disc), and thrombocytosis or polycythemia (elevated platelet count or increased red blood cell mass [8]). The disease can be challenging to diagnose due to the overlapping symptoms with other differential considerations, such as Castleman syndrome. A major distinction between POEMS and Castleman syndrome is the presence of the clonal plasma cell population in POEMS [10].

**Figure 1:**
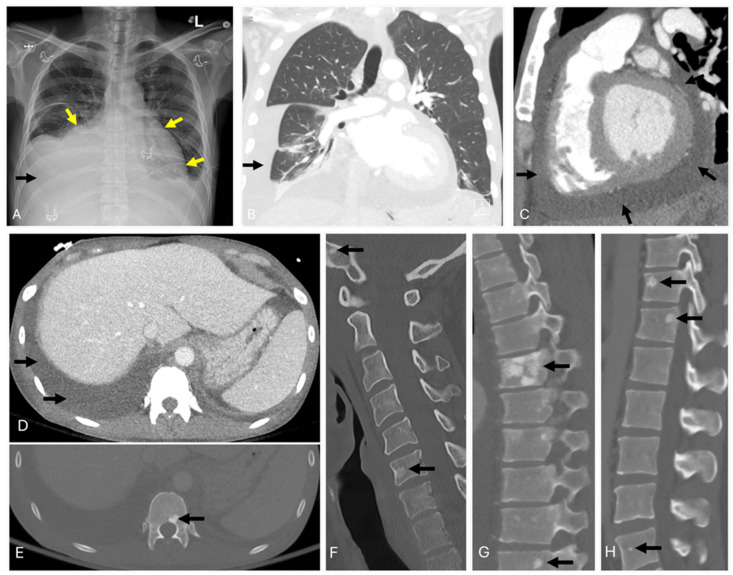
A 40-year-old male with paresthesia. A chest radiograph (**A**) reveals bilateral pleural effusions, with the right greater than the left (black arrows), pulmonary vascular congestion, and pulmonary interstitial edema. The cardiac silhouette is also enlarged (yellow arrows). These findings are confirmed on the contrast-enhanced chest CT (**B**). Additionally, a pericardial effusion is seen (**C**) (black arrows). CT of the abdomen (**D**) shows anasarca and ascites (black arrows). Sclerotic lesions are seen throughout the axial skeleton (**E**–**H**) (black arrows).

Radiological imaging, particularly computed tomography (CT), plays a pivotal role in showcasing characteristic findings such as sclerotic bone lesions and anasarca (Figure 1). Notably, CT is instrumental in detecting sclerotic lesions, which are present in up to 95% of POEMS cases. Whole-body low-dose CT is recommended to detect these osseous lesions since they have a higher detection rate compared with the traditional radiographic skeletal survey. Further, fluorodeoxyglucose positron emission tomography (FDG-PET) may be helpful in the workup to exclude extraosseous sites of malignant plasma cell clones; however, PET has no clear advantage over conventional CT in detecting the classic osteosclerotic lesions seen in POEMS [11,12]. Importantly, the sclerotic lesions of POEMS are often photopenic with no substantial FDG metabolic activity.

### 3.2. AL Amyloidosis

Light chain (AL) amyloidosis is considered to be an independent entity from MGCS; however, it is discussed in this review given that it is an important differential in the workup of monoclonal protein disorders that do not meet the criteria for multiple myeloma [7]. AL amyloidosis is a systemic disorder characterized by low-burden clonal bone marrow plasmacytosis, producing insoluble misfolded light chain fibrils. These insoluble fibrils form beta-pleated sheets that deposit in tissues and organs, which are highly cytotoxic [13,14], resulting in high mortality and morbidity without a timely diagnosis. AL amyloidosis notably affects the heart, kidneys, gastrointestinal tracts, liver, and nerves [15]. Since AL amyloidosis is a rare disease (incidence of approximately 10 cases per million) [16] and the initial symptoms are varied and nonspecific, the diagnosis may not be made until 1 year after symptom onset in some cases. This emphasizes the need for increased clinical suspicion and the evaluation of a monoclonal protein in patients with unexplained symptom constellations [17,18,19]. Along with a syndrome suggestive of AL amyloidosis, the definitive diagnosis of AL amyloidosis entails confirming the presence of amyloid with Congo red evaluation of involved or proxy tissue, subtyping of the amyloid with the appropriate technique to demonstrate light chain subtype and confirmation of an underlying clonal plasma cell process [20,21].

**Figure 2:**
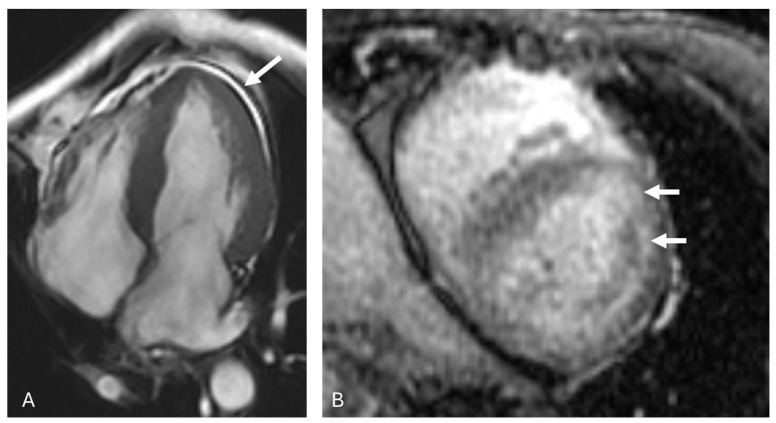
A 66-year-old female with AL amyloidosis. Diffusely thickened left ventricular walls (>17 mm), pericardial effusion (**A**) (arrow) with patchy biventricular subendocardial delayed enhancement (**B**) (arrows).

Radiologic modalities play an important role in the diagnosis of AL amyloid. Overall, 70% of patients present with cardiac involvement, and endomyocardial biopsy is the gold standard. However, 2D echocardiogram and cardiac magnetic resonance imaging (MRI) are important noninvasive techniques for assessing cardiac involvement with respect to the measurement of the ventricular wall and interventricular septum thickness (Figure 2). In terms of an echocardiogram, biventricular wall thickening and biatrial enlargement are highly suspicious imaging features [22]. MRI is sensitive in differentiating AL amyloid from other infiltrative cardiomyopathies. Late gadolinium enhancement is a key feature in establishing this diagnosis. In cardiac amyloidosis, gadolinium contrast tends to accumulate in the amyloid deposits within the myocardial tissue. The pattern of enhancement can be global or patchy and is often seen in both subendocardial and transmural regions. This differs from other cardiomyopathies, where the enhancement is usually confined to the subendocardial region or follows a specific coronary artery distribution. Another common pattern of enhancement is global subendocardial enhancement, in which the enhancement involves the subendocardial layer globally rather than being localized to a specific segment. In addition, the presence of amyloid deposits leads to an increased myocardial extracellular volume, which can be quantified using T1 mapping techniques before and after contrast administration. An elevated extracellular volume is indicative of extensive amyloid infiltration. Further native (non-contrast) T1 values are typically elevated in cardiac amyloid because of amyloid fibril deposits. T2 mapping can sometimes show increased values, reflecting edema or inflammation. Physiological assessment with cardiac MRI can reveal the myocardial strain and restrictive cardiomyopathy. Finally, thickened myocardial walls as a result of amyloid infiltration, often associated with a preserved or slightly reduced left ventricular ejection fraction, can be seen. Following treatment, the improvement or resolution of these findings suggests a positive response to therapy. Technetium pyrophosphate (PYP) scanning aids in differentiating AL amyloidosis from other conditions, such as transthyretin amyloidosis (ATTR), and guides clinical management [23]. Perugini grading is used to assess cardiac involvement with ATTR [24]. Grade 2 or 3 uptake is suggestive of ATTR; however, it is important to note that AL amyloid may demonstrate a degree of uptake on PYP scanning, and a positive PYP scan does not completely exclude AL amyloid [23]. Further, scintigraphy with other agents, including ^99m^Tc-hydroxymethylene diphosphonate and ^99m^Tc-3,3-diphosphono-1,2-propanodicarboxylic acid, are useful in imaging and quantifying the burden of cardiac amyloid [25,26]. Importantly, diagnostic expertise in interpreting these images is crucial to avoid misdiagnosis. The typing of the amyloid is absolutely essential to make the correct diagnosis, as plasma cell-directed treatments for AL amyloidosis differ remarkably from treatments for ATTR. Liquid chromatography tandem mass spectrometry is the current gold standard technique, but immunoelectron microscopy or immunohistochemistry are other options for amyloid typing [20]. Concomitant AL and ATTR amyloid may occur, which may further confound the diagnosis, reinforcing the need for amyloid typing [27,28,29,30].

**Figure 3:**
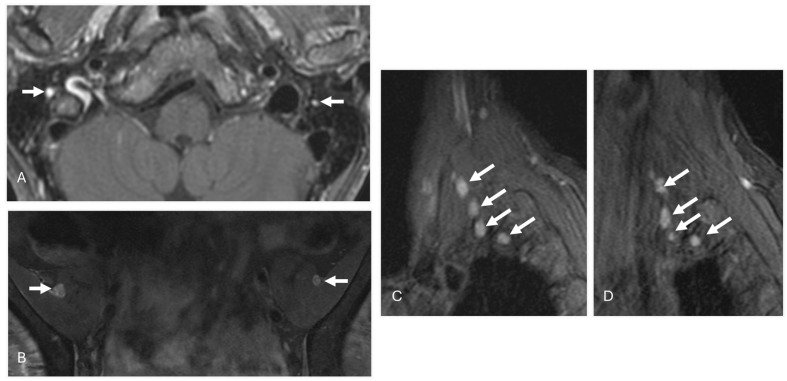
A 70-year-old female with chronic parasthesias and AL amyloidosis. Thickening and enhancement of the right facial nerves (**A**) (white arrows). Similar thickening and increased signal intensity of the femoral nerves (**B**) (white arrows) and the nerves of the brachial plexus bilaterally (**C**,**D**) (white arrows).

**Figure 4:**
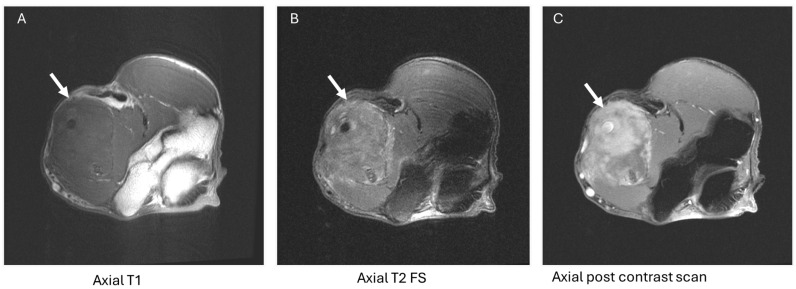
A 63-year-old male with left upper extremity swelling. MRI reveals a T1 hypointense ((**A**), arrow), T2 hyperintense ((**B**), arrow) mass with heterogenous enhancement ((**C**), arrow) encasing the neurovascular bundle within the anterior compartment of the left arm. Mass and bone marrow biopsies confirm the diagnosis of amyloidoma and monoclonal gammopathy.

**Figure 5:**
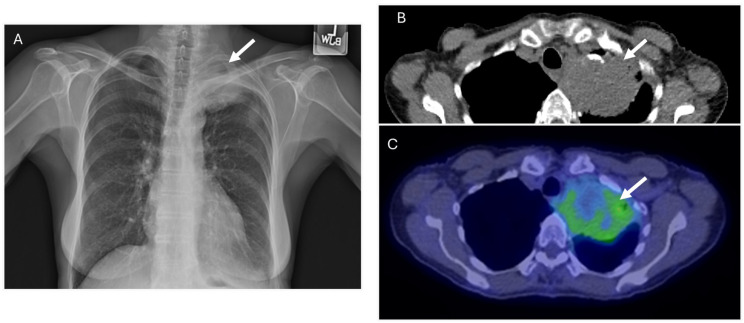
A 76-year-old female with left upper lung mass seen on radiograph ((**A**), arrow) taken after a bicycle accident. This was confirmed as a pulmonary mass on CT ((**B**), arrow) and with internal and peripheral FDG activity on FDG-PET imaging ((**C**), arrow). Percutaneous biopsy revealed pulmonary amyloidoma.

Extracardiac manifestations of amyloidosis involve the peripheral nerves. AT MRI, these are characterized by nerve thickening and increased signal intensity with fluid-sensitive sequences (Figure 3). Finally, amyloid deposits can present as soft tissue masses throughout the body, referred to as amyloidomas (Figure 4 and Figure 5).

### 3.3. Distal Acquired Demyelinating Symmetric Neuropathy with M Protein (DADS-M)

Formerly referred to as MGUS with associated peripheral neuropathy, DADS-M manifests as symmetrical peripheral neuropathy that predominantly affects the distal limbs while sparing the central nervous system [7,31]. Clinically, it presents as sensory disturbances, such as numbness, tingling, or burning sensations, and may include distal muscle weakness. The symptoms typically have a chronic progressive course, worsening over months to years, with sensory symptoms predominating and occasional sensory ataxia due to large fiber sensory loss. Immunoglobulin M (IgM) is frequently implicated, although IgA and IgG variants exist with favorable prognosis [32,33,34]. The cause is IgM deposition, and it is anti-myelin-associated glycoprotein (MAG), identified in 50–70 percent of cases, or other gangliosides [35].

Electrophysiological studies reveal evidence of demyelination and reduced sensory nerve action potentials. Laboratory criteria include the detection of monoclonal immunoglobulin, often IgM, in the serum or urine through immunofixation electrophoresis or serum protein electrophoresis with serum immunofixation confirming the presence and type of monoclonal protein. Additional supporting criteria may include the presence of anti-myelin-associated glycoprotein antibodies, often found in patients with IgM monoclonal gammopathy and DADS neuropathy, and elevated cerebrospinal fluid (CSF) protein without pleocytosis.

**Figure 6:**
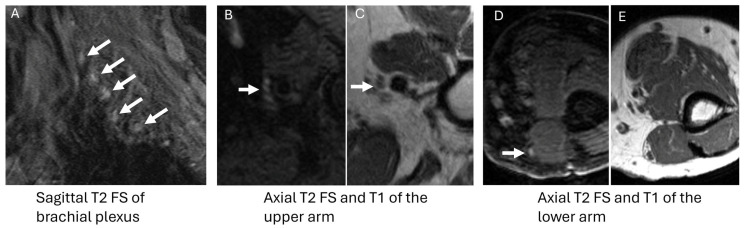
A 75-year-old male with a history of IgM monoclonal gammopathy presents with progressive numbness, paresthesias, and weakness in his hands. MRI showed diffusely increased T2 signal with multifocal areas of neural thickening in the brachial plexus ((**A**), arrows) and peripheral nerves of the upper arm ((**B**–**E**), white arrows). Left median nerve biopsy showed increased demyelination with inflammatory cells, suggesting an immune-mediated demyelinating neuropathic process.

Diagnosis is challenging and primarily exclusionary, necessitating the exclusion of alternative causes of peripheral neuropathy such as diabetes, vitamin deficiencies, toxin exposure, or inherited neuropathies that could exhibit similar symptoms. Importantly, other peripheral neuropathies associated with monoclonal proteins, such as AL amyloidosis and POEMS, should be excluded since therapeutic approaches and prognosis vary [36]. Radiological imaging is nonspecific, and typical imaging findings of peripheral neuropathies are seen via MRI. These include nerve thickening and abnormal fascicular signal intensity with or without nerve enhancement (Figure 6). In addition, sequelae of denervation changes, such as muscular edema-like signals, can be seen.

### 3.4. Chronic Ataxic Neuropathy, Ophthalmoplegia, IgM Paraprotein, Cold Agglutinins, and Disialosyl Antibodies (CANOMAD)

CANOMAD is a rare autoimmune neuropathy characterized by chronic progressive ataxia due to sensory neuropathy, ophthalmoplegia, and peripheral neuropathy, with symptoms such as gait instability, difficulty with fine motor tasks, double vision, and numbness or tingling in the extremities. It is not uncommon to encounter cases without the full spectrum of pathology; therefore, the syndrome may be referred to as CANDA—chronic ataxic neuropathy with disialosyl antibodies [37]. Monoclonal IgM antibodies target gangliosides that contain disialosyl groups [38]. Anti-MAG is negative in comparison with DADS-M and other immunoglobulin-related peripheral neuropathies [39].

The diagnostic criteria include the detection of monoclonal immunoglobulin, typically IgM, in the serum through serum protein electrophoresis or immunofixation electrophoresis, the presence of cold agglutinins (autoantibodies that cause red blood cells to clump together at low temperatures), and anti-disialosyl ganglioside antibodies, which can be identified using enzyme-linked immunosorbent assays or other immunological tests. Additional supportive features include elevated cerebrospinal fluid protein levels without pleocytosis and nerve conduction studies that may show demyelinating or axonal neuropathy. To confirm the diagnosis, it is essential to exclude other causes of similar neuropathies, such as chronic inflammatory demyelinating polyneuropathy, Miller Fisher syndrome, and other systemic diseases or infections that can cause neuropathy.

**Figure 7:**
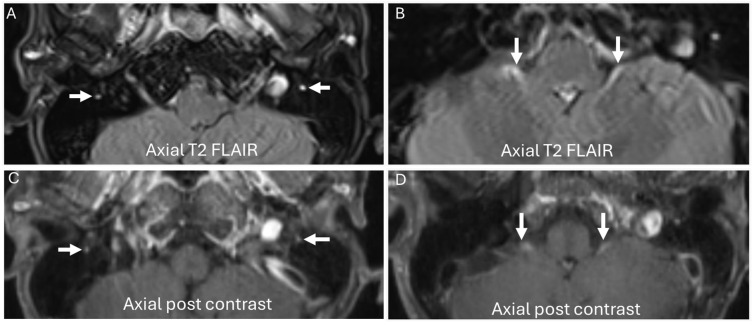
A 57-year-old male with a history of IgM monoclonal gammopathy presents with worsening bulbar dysfunction and respiratory weakness. MR brain reveals symmetric increased signal intensity and enhancement of both oculomotor and facial nerves ((**A**,**C**), arrows) as well as spinal accessory nerves ((**B**,**D**), arrows).

The diagnosis is intricate, often delayed, and with a considerable gap of up to four years between the onset of symptoms and confirmation [39]. Unfortunately, by the time of diagnosis, most patients may have developed significant disability. Early suspicion is crucial in MGUS patients presenting with pertinent symptoms, necessitating a multifaceted diagnostic approach that is mostly reliant on laboratory assessments. Imaging plays a limited role in the diagnosis of CANOMAD, given the lack of specific features. At MRI, the involved cranial nerves are thickened with increased signal intensity on T2-weighted imaging and mild nerve enhancement (Figure 7).

## 4. Monoclonal Gammopathy of Renal Significance (MGRS)

The term MGRS was introduced by the International Kidney and Monoclonal Gammopathy Research Group (IKMG) in 2012. MGRS is an umbrella term for clonal plasma cell disorders that do not meet the criteria for multiple myeloma (bone marrow plasmacytosis <10%) but are associated with nephrotoxic monoclonal immunoglobulin [2,3]. The monoclonal immunoglobulins elicit their toxic effects through diverse mechanisms, and kidney biopsy is critical for distinguishing among the various patterns of injury and excluding cast nephropathy as the cause of renal impairment [40,41]. MGRS conditions are classified based on kidney biopsy findings based on the deposition patterns of the monoclonal immunoglobulin deposition on electron microscopy [42]. Immunoglobulin deposition associated with MGRS is further categorized according to the pattern of deposition: organized and non-organized types.

MGRS lesions with non-organized deposits include Monoclonal Immunoglobulin Deposition Disease (MIDD), which comprises light chain deposition disease (LCDD), heavy chain deposition disease (HCDD), and light and heavy chain deposition disease (LHCDD). These conditions may have extra-renal manifestations [42,43]. Proliferative Glomerulonephritis with Monoclonal Immunoglobulin Deposits (PGNMID) is renal-limited and marked by non-organized monoclonal immunoglobulin deposits in the glomeruli presenting with renal impairment and nephrotic range proteinuria [42,44]. C3 Glomerulopathy with Monoclonal Gammopathy is another MGRS condition with non-organized deposits where monoclonal immunoglobulins dysregulate the complement system, leading to C3 complement component deposits in the glomeruli and subsequent kidney damage [42].

MGRS conditions with organized deposits include light chain proximal tubulopathy, which presents with evidence of tubular dysfunction with or without Fanconi syndrome [45]. Microscopy demonstrates the inclusion of light chains in the proximal tubule cells [46]. Cryoglobulinemic Glomerulonephritis is another MGRS with organized deposits; cryoglobulins precipitate at low temperatures, causing inflammation and damage in the kidneys [42]. Immunotactoid Glomerulopathy is another form of rare organized MGRS lesions associated with glomerular microtubular deposits resulting from monoclonal gammopathy [47]. Renal lesions for light chain (or heavy chain) amyloidosis are considered to be organized MGRS lesions and have been discussed previously [42].

Imaging plays a critical role in ruling out multiple myeloma or other B-cell/plasma cell disorders in patients suspected of MGRS. Though renal prognosis is worse, the overall survival for MGRS is superior to patients with multiple myeloma; therefore, distinguishing between the conditions is important in the diagnostic pathway [4]. Advanced whole-body imaging with CT, MRI, or PET can be used to identify sites of localized disease or plasmacytoma that can then be biopsied.

## 5. Cutaneous Manifestations of Monoclonal Gammopathy of Clinical Significance (MGCS)

MGCS with predominant cutaneous manifestations may be termed monoclonal gammopathy of cutaneous or dermatologic significance. Schnitzler syndrome is one such example. Cutaneous involvement associated with an underlying monoclonal protein disorder may also present as part of a broader multisystemic syndrome, such as Cryoglobulinemia and POEMS syndrome. While there is no clear role of imaging in their diagnoses, a few of these cutaneous manifestations of MGCS have been discussed. Others, like TEMPI syndrome and Clarkson’s disease, have no pathognomonic imaging findings.

### 5.1. Type 1 Cryoglobulinemia

Type 1 Cryoglobulinemia is characterized by monoclonal immunoglobulin, unlike types 2 and 3, which are commonly associated with polyclonal immunoglobulins linked to infections [48]. It may present concomitantly with monoclonal gammopathy of undetermined significance (MGUS), B cell lymphomas, or malignant disorders of B cells or plasma cells [49]. Cutaneous symptoms may include purpura, livedo reticularis, cold urticaria, and ulcers in severe cases, often triggered by cold temperatures. Additionally, the nervous system can be affected, primarily resulting in peripheral neuropathy, with rare occurrences of central nervous system involvement.

### 5.2. Necrobiotic Xanthogranuloma (NXG)

NXG is associated with lymphoproliferative or plasma cell disorders. It typically presents as papules of various colors (yellow or dark red), which may coalesce to form larger plaques, nodes, telangiectasia, or ulcers. Extracutaneous involvement, though uncommon, can lead to significant morbidity or mortality, affecting organs such as the liver, spleen, and heart [50].

**Figure 8:**
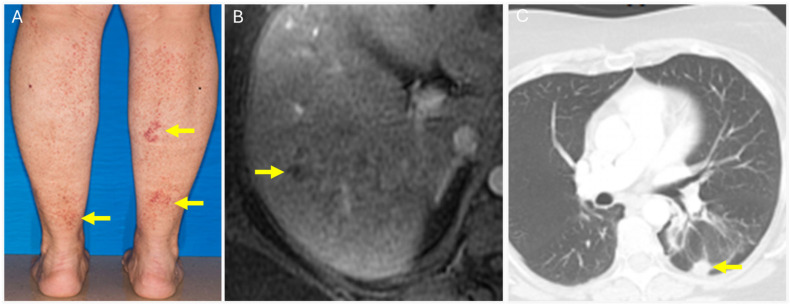
A 51-year-old female with a history of IgG kappa monoclonal gammopathy presented with persistent and increasingly tender nodules of the posterior calves (**A**, yellow arrows). Skin biopsy showed NXG. MR abdomen (**B**, yellow arrow) revealed hepatic infiltration with innumerable non-enhancing tiny nodules, and liver biopsy showed NXG. Chest CT (**C**, yellow arrow) found a left lower lobe nodule, and lung biopsy showed NXG.

**Figure 9:**
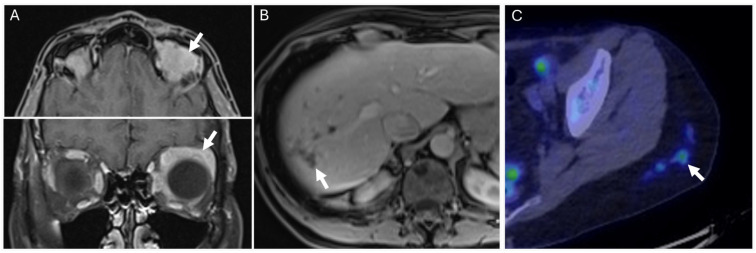
A 59-year-old female with rapid growth of an eyelid lesion. MRI of the orbit (**A**, arrows) with a plaque-like mass in the superomedial left preseptal orbit. Biopsy was consistent with NXG. Further workup revealed transaminitis and MRI abdomen (**B**, arrow) revealed linear and nodular areas of hypoenhancement in the right lobe of the liver. Biopsy was positive for NXG. PET CT revealed additional nodules, such as in the subcutaneous soft tissues of the left posterolateral thigh (**C**, arrow) with SUV max 6.1, which were also biopsied and positive for NXG. The patient was also found to have IgM monoclonal gammopathy.

Diagnosis relies on typical yellow skin lesions, histopathological-confirmed, as a major criterion, with monoclonal gammopathy and periorbital distribution as minor criteria [51]. Following diagnosis, investigations to assess extracutaneous involvement are warranted, with radiological examinations, such as CT (with or without PET) for thoracic lesions and MRI for orbital lesions, proving useful for lesion detection. (Figure 8 and Figure 9).

### 5.3. Schnitzler Syndrome

Schnitzler syndrome presents with chronic urticarial rash, arthralgia, bone pain, chronic intermittent fever, and bone pain with skeletal hyperostosis. IgM is commonly associated with this syndrome, though IgG may also be found in rare cases. Diagnosis is based on the Strasbourg criteria, which consider rash and monoclonal gammopathy as obligatory criteria and other disease features as minor criteria [52]. In addition to the presence of both major criteria, at least two minor criteria are required. These include recurrent fever, bone pain with osteosclerotic lesions, arthralgia or arthritis, hepatomegaly or splenomegaly, lymphadenopathy, elevated inflammatory markers (e.g., CRP, ESR), or leukocytosis. These criteria aim to distinguish Schnitzler syndrome from other conditions presenting with similar symptoms, necessitating thorough exclusion of infections, malignancies, and other autoimmune or autoinflammatory diseases. In 59% of cases, bone lesions are seen [53]. On MRI, these are reported to have characteristic cortical thickening and intramedullary T2 hyperintense lesions [54].

## 6. Imaging Considerations

The role of imaging in the workup of monoclonal gammopathies varies widely, reflecting the diverse nature of these conditions. For malignant and premalignant monoclonal gammopathies, such as MGUS, plasmacytomas, or multiple myeloma, the value of imaging is clear, and societal guidelines on imaging recommendations exist to guide clinical practice. MRI is universally accepted as the gold standard for lesion detection due to its superior contrast and tissue resolution. Additionally, molecular imaging with PET is the preferred modality for determining changes in disease activity, as it provides metabolic information that complements the anatomical detail provided by MRI. CT, on the other hand, is the test of choice for identifying bone destruction, offering detailed evaluations of bone integrity and the presence of lytic or sclerotic lesions [1,55].

However, unlike malignant or premalignant conditions, there are no clear guidelines for the majority of MGCS. As a result, the approach to imaging in these cases is highly disease-specific and guided primarily by symptomatology. A summary of our assessment of the imaging features of MGCS is included in Table 1. For neuropathic MGCS diseases, MRI plays a critical role in evaluating the nerves for abnormalities in size and morphology. MRI is also crucial for identifying associated neural pathology, such as muscular denervation, due to its superior contrast resolution and ability to visualize soft tissues in detail. Although monoclonal disease activity specifically involving nerves can be detected on MRI with the use of intravenous contrast enhancement, molecular imaging with PET most clearly depicts such activity, making it the preferred method in these cases. In fact, PET/MRI, which combines the superior anatomical resolution of MRI with the metabolic imaging capabilities of PET, may be ideal for the workup of MGCS with suspected nerve involvement. In addition to the detection of lesions, imaging can play a role in assessing response to therapy and for relapse assessment.

CT scans are particularly helpful for specific tasks, such as the detection of lytic or sclerotic lesions characteristic of certain diseases. For instance, sclerotic lesions in the setting of POEMS syndrome (Polyneuropathy, Organomegaly, Endocrinopathy, M-protein, and Skin changes) can be effectively identified using CT. Additionally, CT is useful for excluding lytic lesions that would indicate a malignant condition, such as multiple myeloma. Furthermore, CT is adept at detecting associated pathology, including pleural effusions, pericardial effusions, ascites, pulmonary nodules, and abdominopelvic mass lesions. The relative ease of access to CT, compared to MRI and PET, as well as the shorter scan times and relative comfort for patients, contribute to its widespread use in clinical practice.

Cardiac MRI for the workup of cardiac involvement in amyloid is a well-established imaging modality with specific recommendations. A near-definitive diagnosis regarding the presence or absence of cardiac amyloid can often be made based on MRI findings. Similar to CT, cardiac MRI can also detect associated sequelae, such as pleural effusions or intrathoracic mass lesions. However, there are drawbacks to using MRI, including limited access to cardiac MRI in some imaging centers and contraindications such as certain implanted devices and orthopedic hardware. Additionally, the presence of implanted cardiac devices, which are common in patients with cardiac symptoms, can create artifacts that limit the utility of cardiac MRI for detecting subtle findings.

Although renal involvement from MGCS represents a significant component of the clinical presentation in several of these conditions, renal imaging with MRI, CT, or ultrasound is not particularly helpful or additive in establishing a diagnosis. However, the sequelae of renal failure and poor renal function, including pleural effusions, ascites, and anasarca, are readily visualized on imaging, and their presence can suggest underlying renal pathology. Additionally, end-stage renal disease can be seen on imaging as renal parenchymal atrophy, although the diagnosis is typically made before this stage based on clinical and laboratory findings.

Finally, there is little, if any, role for imaging in the workup of cutaneous involvement in MGCS. Cutaneous lesions suggestive of pathology are readily detected on physical examination, making additional imaging of the skin unnecessary for diagnosis. However, associated imaging findings, such as extracutaneous involvement of disease in solid organs and subcutaneous tissues, as seen in the case of NXG, can be detected and may aid in diagnosis or guide tissue sampling for histopathological confirmation.

## 7. Conclusions

MGCS and related disorders present with diverse and enigmatic clinical manifestation. A multidisciplinary approach is required for diagnosis and management. Radiological imaging plays a crucial role in characterizing several of these diseases and aids in narrowing differential diagnoses.

## Figures and Tables

**Table 1:** Summary of pertinent imaging findings seen in monoclonal gammopathies of clinical significance.

Disorder	Radiological Diagnostic Findings
POEMS Syndrome	CT detects sclerotic bone lesions and anasarca. PET-CT excludes extraosseous sites of malignant plasma cell clones.
AL Amyloidosis	Echocardiogram detects biventricular wall thickening and biatrial enlargement. Cardiac MRI detects myocardial thickness and is sensitive in differentiating amyloidosis from other infiltrative disorders. Pyrophosphate scan differentiates AL amyloidosis from ATTR amyloidosis. CT detects extracardiac accumulation of amyloid as masses in various organs and identifies cardiomegaly. MRI identifies accumulation of amyloid as masses and infiltration in various organs.
DADS-M	MRI identifies signs of peripheral neuropathy and denervation as well as response to treatment.
CANOMAD	MRI identifies signs of neuropathy in affected cranial nerves.
MGRS	PET, CT or MRI to exclude multiple myeloma or plasmacytomas.
Monoclonal gammopathy of cutaneous significance	Imaging has a limited role. In NXG, whole-body imaging with CT, MRI or PET imaging identifies extracutaneous sites of involvement.

AL: light chain amyloidosis; ATTR: amyloid transthyretin; MGRS: monoclonal gammopathy of renal significance; NXG: necrobiotic xanthogranuloma.

## Data Availability

No new data were created or analyzed in this study. Data sharing is not applicable to this article.
